# Prevalence and Determinants of Suicide Ideations Among Students of Selected Tertiary Learning Institutions in Lusaka, Zambia: A Cross‐Sectional Study

**DOI:** 10.1002/hsr2.72972

**Published:** 2026-08-09

**Authors:** Josephine Nkhowani, Dennis Ngosa, Mwiche Musukuma, Patrick Kaonga, Choolwe Jacobs

**Affiliations:** ^1^ Department of Epidemiology and Biostatistics, School of Public Health University of Zambia Lusaka Zambia; ^2^ Women in Global Health Zambia Lusaka Zambia; ^3^ Analysis Unit Center for Infectious Diseases Research in Zambia Lusaka Zambia

**Keywords:** hopelessness, mental health, suicide ideations, university students

## Abstract

**Background and Aims:**

The transition into university life can be overwhelming for many individuals, as this stage presents new academic, personal, and social change and expectations which can significantly influence a student mental well‐being. Failure to cope, low social connectedness and lack of hopefulness about the future has the potential to tip students to suicide ideations. The prevalence and factors associated with suicide ideations among university students is not well investigated in Zambia. Therefore, this study set out to investigated the prevalence and factors associated with suicide ideations among students of selected universities in Lusaka, Zambia.

**Methods:**

This was an cross‐sectional study conducted at two universities in Lusaka from July to October, 2023 among undergraduates aged 18–29 years. Probability proportional to size was used to determine needed sample size. Participants were pragmatically sampled and a structured questionnaire was used to collect data. Suicidal ideations within the past twelve months was taken as outcome variable. Unadjusted and adjusted logistic regressions were performed at 95% confidence interval.

**Results:**

Out of the total 384 participants, 71% (272) from University of Zambia and 29% (112) from University of Lusaka. Slightly over half (54%) were females and majority (90%) were aged 18‐24 years. The prevalence of suicidal ideations within the past 12 months was reported at 33.9% [95% CI: 29.3–38.8]. Factors such as drug/alcohol consumption (AOR = 1.79, CI: 1.08–2.97), depressive symptoms (AOR = 2.17, CI: 1.30–3.64), feelings of hopelessness (AOR = 3.89, CI: 1.47–10.37), and lack of supportive families (AOR = 3.19, CI: 1.45–6.91) were significantly associated with increased odds of suicide ideations while being male was protective compared to being female (AOR = 0.61; 95% CI: 0.38–0.97).

**Conclusion:**

The study underscores existence of prevalent suicide ideations among university students, emphasizing the critical importance of addressing mental health challenges among university students.

## 1 Introduction

“Why suicide?”, cries of friends and loved ones of the deceaseds dead by suicide. Filled with feelings of frustration, guilt and self blame thinking there is something they should have or could have done to prevent their loved one from ending their own life [1, 2].

Suicide is a major public health issue, with long‐lasting and severe effects on families, friends, and communities [3]. The World Health Organization's Gap Action Program of 2008 recognized suicide as a priority for targeted attention and action [4]. Globally, suicide leads to more than 800,000 deaths per year, with a considerable number occurring among young individuals aged 15–29 years [4, 5, 6] an increasingly active subgroup of this young population being university students [7].

The issue of suicide among young people, particularly university students, is a concerning public health problem [8], with a lifetime prevalence of suicide ideation reported at 46.4% [9]. Suicide ideations are defined as thoughts, thinking about, considering, or planning to take one's own life. Suicide ideations and attempts are strong predictors of suicidal deaths [10]. These ideations encompasses both passive (desire to die without a plan) and active (desire to die with a plan) forms, with both strongly predicting suicidal deaths [11]. Suicide ideations are important and occur far more frequently prior to any suicide attempt, with death by suicide being the tip of the iceberg [12, 13]. A study conducted among students in Sub‐Saharan Africa, reported Zambia to have the highest prevalence of suicide ideations at 31.9% [14]. The transition into university life can be overwhelming for many individuals, as this period marks a significant psychological, social, and environmental change. Transitioning into university life frequently involves moving away from ones parental home, and a change in daily routines. These adjustments can make it difficult for students to adapt to academic life, increasing the likelihood of experiencing depressive emotions such as loneliness and despair [7, 12, 13, 15, 16, 17]. The overwhelming cognitive and social change characterized by negative beliefs about the future and perceived inability to cope has the ability to make students experience hopelessness, which is a pessimistic outlook on the future and is associated with suicide ideations [18].

A transnational study conducted in 19 colleges across 8 countries in Africa, Europe, South America, and North America reported a lifetime prevalence of 32.7% [10] of suicide ideation among the students. Previous studies have reported suicide ideations to be prevalent among university students that have depressive symptoms [11, 19], that are hopeless about the future [20] and those that consume psychoactive substances like alcohol [21, 22]. Additionally, other studies have reported factors such as not enrolling in desired program of study in the desired [23], being a female student, family suicide history [11] and having poor social support [24] to be associated with suicide ideations among university students.

Unlike other health outcomes, studying and preventing suicide poses unique challenges due to its retrospective nature and the complexity of determining intent [25, 26]. Investigators instead often study suicidal thoughts (ideation) or behaviors as proxies for suicide because they are strongly related to suicide and occur far more frequently [27]. Research on suicidal ideations in most developing countries among students is limited due to resource constraints and the stigma surrounding suicide in certain countries within the region [28, 29]. Conducting studies on suicide ideations, like this particular one bridges the knowleges gap and provide valuable insights in understanding suicide ideations among students. Therefore, this study aimed to assess the prevalence and factors associated with suicide ideation among undergraduate students in Lusaka, Zambia.

## 2 Methodology

### 2.1 Study Design, Setting, and Population

This was a cross‐sectional study conducted between July and October, 2023, in two selected universities in Zambia; the university of Zambia (UNZA) and the university of Lusaka (UNILUS). The university of Zambia was selected for its large student population as it is the largest government owned university in Zambia, while the university of Lusaka was chosen for its status as the largest private owned university in Zambia.

### 2.2 Participants

Undergraduate students aged 18–29 years and registered for the 2023 academic year were eligible to participate in the study. Students that deffered their studies to the next year or had not yet registered for the 2023 academic year were excluded from the study.

### 2.3 Sample Size

Using the prevalence sample size formula for an infinite population, the expected proportion of suicidal ideation in the past 12 months (P) was set at 0.40 (40%) [14, 30] at 95% confidence level, (Z) was set at 1.96 and margin of error (d) set at 0.05 (5%), the minimum sample size was 369 students. Taking into account 5% non‐response rate the study had a target sample size of 388 Students. This sample size was then proportionately divided between the two selected universities. The university of Zambia had 24,198 students registered for the 2023 academic year and the university of Lusaka had 10,000 students. This gave a total of 113 students to be sampled from the University of Lusaka main campus and 275 students to be sampled from the University of Zambia main campus.

### 2.4 Sampling Technique

A pragmatic approach was used to sample the students based on convenience, allowing for practical and efficient data collection. Pragmatic sampling involves selecting participants based on accessibility and feasibility rather than random selection, making it suitable for studies with limited time and resources. Students were sampled as they were leaving various campus locations such as school libraries, cafeterias, lecture rooms, university hostels, and surrounding boarding houses. Additionally, pragmatic sampling enabled research assistants to collect data in real‐world settings, reflecting the natural behavior and environment of the participants [31].

### 2.5 Data Collection Procedure

A sturctured questionnaire build in kobo collect was used as the data collection tool. After collecting participant informed consent, data collection was done either physically or via phone call for students who said were busy but wanted to take part in the study at a later time. Students who agreed to take part in the study were administered a structured questionnaire.

### 2.6 Data Collection Tools

#### 2.6.1 Suicide Ideations

The suicide behavior questionnaire‐revised (SBQ‐R) was used in the study to collect data on the prevalence of suicide ideations among the students. The SBQ‐R tool consists of four items. The first item assesses lifetime suicidal thoughts and attempts, the second item addresses the frequency of suicidal thoughts in the past 12 months, the third item is about the communication of suicidal thoughts or plans to others and the fourth item focuses on the future risk of suicide attempts [32]. The main variable for this study was the second item which assess suicide ideations in the past 12 months. The frequency of suicide ideations was measured on a five‐point likert scale of either Never or Rarely (1 time) or Sometimes (2 times) or Often (3–4 times) or Very Often (5 or more times). Ideation frequency was then coded as a dichotomous variable with 0 for the students who didn't experience suicide ideations in the past 12 months (students whose response was never) and 1 for the students that selected either (Rarely or Sometimes or Often or Very Often). The (SBQ‐R) tool as been reported to have a Cronbach's alpha of between 0.76 and 0.88 which indicates a good internal consistency and reliability of tool items [33].

#### 2.6.2 The Depression Module

The Patient Health Questionnaire (PHQ‐9) which is a self‐report measure for the assessment of depressive symptoms was used to collect data on depression in the study. The PHQ‐9 screens for depressive symtoms within the last 2 weeks,. On a 4‐point‐Likert‐Scale ranging of 0 (not at all), 1 (several days), 2 (more than half the days) and 3 (nearly every day).

The PHQ‐9 included items: Little interest or pleasure in doing things and Feeling down Trouble falling or staying asleep, or sleeping too much and Feeling tired or having little energy etc. However, the 9th item evaluates passive thoughts of death or self‐injury and is often used to screen depressed patients for suicide risk was not included in our calculation [34] to avoid direct conceptual and statistical overlap with the outcome. The total score was calculated from the eight remaining items. The total score of the eight items ranges from 0 to 27. Using a cut‐off score of 10, the total score was then categorized into two categories. For score ≥ 10 detecting the presence of clinically relevant symptoms and was coded as 1 and for scores < 10 indicating the asbsence of depressive symptoms and was coded 0 [35].

#### 2.6.3 Hopelessness

Hopelessness was assessed using the 7th item of the Beck Hopelessness Scale which states “My future seems dark to me” with responses True or False and was code as 1 and 0 respectively. Studies have shown that this statement is sufficient enough to assess hopelessness, it is ideal for summarizing the construct under investigation [36]. This has also been supported by other studies whose results showed that this item had the highest item‐residue correlation of (r = 0.75) [37, 38].

#### 2.6.4 Academic Performance

Academic Performance Scale was used to assess academic performance [39]. The total score has an internal consistency of 0.89 and a test‐retest reliability of 0.85 [40]. The scale has eight items which are positively phrased on a 5 point‐Likert scale of strongly agree, agree, neutral, disagree, and strongly disagree. This continuous variable was then categorized and coded using the scores and parameters of 0–8 for failing performance, 9–16 for poor performance, 17–24 for moderate performance, 25–32 for good performance and 33–40 for excellent performance.

### 2.7 Data Analysis

Data was analyzed using STATA version 16.0 (StataCorp, College Station, TX, USA). Descriptive statistics such as frequencies and percentages were used to summarize the study participants characteristics. The prevalence of suicidal ideation in the past 12 months was calculated by taking the number of students who had a “Yes” answer and dividing it by the total number of study participants as the denominator using the SBQ‐R tool. The proportion of people who had suicidal ideation in the past 12 months was then compared across social‐demographic and academic characteristics of students using the Chi‐square and Fisher's exact tests based on the assumptions of each test.

Taking suicidal ideation in the past 12 months as the outcome variable, which was coded = 1 if “Yes”, otherwise = 0, logistic regression was performed at adjusted and unadjusted levels. Machine‐lead stepwise and investigator‐lead stepwise logistic regressions were used at adjusted analysis. Robust standard errors were used when taking care of clustering by the university. The pseudo‐R2 and Bayesian Information Criteria (BIC) were used to choose the best‐fit model, of which the investigator lead stepwise regression was settled for, as the best‐fit model. All forms of analysis in this study were done at a 95% confidence interval and robust odd ratios were used to report effect size.

### 2.8 Ethical Considerations

Ethical approval was sought from the University of Zambia Biomedical Research Ethics Committee and the study was approved REF. 3929‐2023. Permission to conduct the study was also sought from the University of Zambia and University of Lusaka student registrars. During data collection, each student was given an information sheet for the study, confidentiality was assured to study participants, and any risks or benefits of the study were explained. After which, written informed consent was sought from the study participants. Participants were informed that they were free to stop participation or skip any questions in the study without any consequences. Partcipants that said they needed help immediately where given contact details of a psychosocial counselor from the University Teaching Hospital (UTH) in Lusaka and the suicide toll free help line.

## 3 Results

### 3.1 Descriptive Characteristics of Study Participants

A total of 384 students participated in this study out of the targeted sample of 388. This gave us a response rate of 99% (384/388).

Out of the total participants, 71% (272) where from the University of Zambia while 29% (112) where from the University of Lusaka. More than half (54%)of the participants were female, and the majority 90% (347) were aged 18–24 years. Half (50%, *n* = 192) lived in within the university campus, while others stayed in boarding houses away from the university campus 27% (103). Regarding finances, half 50% (193) of study participants has their fees paid by parents/guardians, while 47% (180) relied on sponsorship/scholarships. The majority 82% (316) of the students came from households where the head of the household was in employment. A quarter 25% (97) of the students reported consuming drugs/alcohol. Additional descriptive statistics are shown in Table 1.

**Table 1:** Socio demographic and academic characteristics of participants.

Characteristics	Learning Institution *n* (%)		Total *n* (%) *N* (384)
	UNILUS *n* (112)	UNZA *n* (272)	
Age (years)			
18–24	101 (90.2)	246 (90.4)	347 (90.4)
25–29	11 (9.8)	26 (9.6)	37 (9.6)
Sex			
Female	52 (46.4)	157 (57.7)	209 (54.4)
Male	60 (53.6)	115 (42.3)	175 (45.6)
Current residence			
Boarding house	48 (42.9)	55 (20.2)	103 (26.8)
Home	61 (54.5)	28 (10.3)	89 (23.2)
School hostel	3 (2.7)	189 (69.5)	192 (50.0)
Romantic relationship status			
Not in a relationship	73 (65.2)	190 (69.9)	263 (68.5)
In a relationship	39 (34.8)	82 (30.1)	121 (31.5)
Drug/alcohol use			
No	72 (64.3)	215 (79.0)	287 (74.7)
Yes	40 (35.7)	57 (21.0)	97 (25.3)
Year of study			
First year	34 (30.4)	67 (24.6)	101 (26.3)
Second to fourth year	78 (69.6)	205 (75.4)	283 (73.7)
Academic performance			
Moderate	7 (5.4)	56 (21.0)	63 (16.1)
Good	86 (76.8)	179 (65.8)	265 (69.0)
Excellent	19 (17.0)	37 (13.6)	56 (14.6)
Academic financial support			
Subsidized/scholarship	11 (9.8)	169 (62.1)	180 (46.9)
Parent/guardian	92 (82.1)	101 (37.1)	193 (50.3)
Partner/spouse (husband/wife)	9 (8.0)	2 (0.7)	11 (2.9)
Living expenses support			
Subsidized/scholarship	1 (0.9)	23 (8.5)	24 (6.3)
Parent/guardian	100 (89.3)	239 (87.9)	339 (88.3)
Partner/spouse (husband/wife)	11 (9.8)	10 (3.7)	21 (5.5)
Occupation of household Head			
Not employed	17 (15.2)	51 (18.8)	68 (17.7)
Employment	95 (84.8)	221 (81.3)	316 (82.3)

Abbreviations: %, percentage; n, sample; UNILUS, University of Lusaka; UNZA, University of Zambia.

### 3.2 Prevalence of Suicide Ideations

The overall prevalence of suicidal ideation within the past 12 months was 33.9% (130) at (95% CI: 29.3%, 38.8%), Table 2 below shows the responses to the second item of the SBQ‐R that was used to calculate this prevalence.

**Table 2:** Suicidal ideation among university students.

Item 2 SBQ‐R: “How often have you thought about killing yourself in the past year?”	Frequency *N* = 384	Percentage (%)
1. Never	254	66.2
2. Rarely (1 time)	57	14.8
3. Sometimes (2 or more)	18	4.7
4. Often (3 or 4 times)	34	8.9
5. Very often (5 or more times)	21	5.5

Abbreviations: %, Percentage; *N*, Sample.

### 3.3 Comparing Suicidal Ideation Across Different Student Characteristics

Findings revealed that there was a statistically significant proportional difference between female students who reported suicide ideations in the past 12 month compared to male students (63.1% vs 36.9%). More students in subsequent years of study had suicidal ideation compared to students in their first year of study (66.9% vs 33.1%). Similarily, the proportion of students who consumed alcohol or drugs that reported suicide ideations was significantly higher compared to those who did not consume alcohol or drugs in the past 12 months (66.9% vs 33.1%).

Furthermore, there was no proportional difference among the number of students who reported suicide ideations at the university of Zambia and the university of Lusaka (64.6% vs 35.4%). The number of students presenting with suicide ideations (31.5%,19.2%, and 49.2%) among those living in boarding houses away from the university campus, students coming from home and those living in a university hostel was not statistically significant respectively. Further reference is in Table 3 below.

**Table 3:** Suicidal ideation across characteristics of University of Zambia and University of Lusaka students, Lusaka Zambia July–October 2023.

Characteristics	Total *n* (%)	Suicide ideation *n* (%)		*p*‐value
		No	Yes	
Overall	384	254 (66.1)	130 (33.9)	
Age group				
18–24	347 (90.4)	228 (89.8)	119 (91.5)	0.58^c^
25–29	37 (9.6)	26 (10.2)	11 (8.5)	
Sex				
Female	209 (54.4)	127 (50.0)	82 (63.1)	0.015^c^ *
Male	175 (45.6)	127 (50.0)	48 (36.9)	
Current residence				
Boarding house	103 (26.8)	62 (24.4)	41 (31.5)	0.23^c^
Home	89 (23.2)	64 (25.2)	25 (19.2)	
School hostel	192 (50.0)	128 (50.4)	64 (49.2)	
Romantic relatioship status				
No	263 (68.5)	176 (67.3)	87 (66.9)	0.64^c^
Yes	121 (31.5)	78 (30.7)	43 (33.1)	
Drug/alcohol use				
No	287 (74.7)	200 (78.7)	87 (66.9)	0.012^c^ *
Yes	97 (25.3)	54 (21.3)	43 (33.1)	
Study year				
First year	101 (26.3)	58 (22.8)	43 (33.1)	0.031^c^ *
Subsequent years	283 (73.7)	196 (77.2)	87 (66.9)	
Academic performance				
Moderate	63 (16.4)	36 (14.2)	27 (20.8)	0.21^c^
Good	265 (69.0)	178 (70.1)	87 (66.9)	
Excellent	56 (14.6)	40 (15.7)	16 (12.3)	
Institution				
University of Lusaka	112 (29.2)	66 (26.0)	46 (35.4)	0.055^e^
University of Zambia	272 (70.8)	188 (69)	84 (64.6)	
Academic financial support				
Subsidy/scholarship	180 (46.9)	130 (51.2)	50 (38.5)	0.044^e^ *
Parent/guardian	193 (50.3)	116 (45.7)	77 (59.2)	
Partner/spouse husband/wife	11 (2.9)	8 (3.1)	3 (2.3)	
Living expenses support				
Subsidy/scholarship	24 (6.3)	16 (6.3)	8 (6.2)	1.00^e^
Parent/guardian	339 (88.3)	224 (88.2)	115 (88.5)	
Partner/spouse husband/wife	21 (5.5)	14 (5.5)	7 (5.4)	
Occupation head				
Not employment	68 (17.7)	44 (17.3)	24 (18.5)	0.78^c^
Employed	316 (82.3)	210 (82.7)	106 (81.5)	
Depressive symptoms				
Not depressed	292 (292)	211 (83.1)	81 (62.3)	< 0.001^c^ *
Depressed	92 (24.0)	43 (16.9)	49 (37.7)	
Hopelessness				
No	367 (96)	249 (98)	118 (91)	0.0018^c^ *
Yes	17 (4)	5 (2)	12 (9)	
Supportive family				
Yes	344 (89.6)	241 (94.9)	103 (79.2)	< 0.001^c^ *
No	40 (10.4)	13 (5.1)	27 (20.8)	
Family suicidal				
No	310 (80.7)	209 (82.3)	101 (77.7)	0.28^c^
Yes	74 (19.3)	45 (17.7)	29 (22.3)	

Abbreviations: %, percentage; c, chi‐square test; e, Fisher's exact test; *n*, frequency; *N*, sample number.

* Statistically significant *p* value at 5% significance level.

### 3.4 Factors Associated With Suicide Ideations

At unadjusted analysis male sex and students who were in subsequent years of studies had statistically significant lesser odds of suicidal ideation in the past 12 months.

The best fit model for adjusted regression had a total of 5 variables. And it was revealed that males had statistically significant lesser odds of sucidal ideation (AOR = 0.61; 95% CI = 0.37, 0.97, *p* = 0.036). On the other hand, students that reported history of substance abuse, depressive symptoms, feeling of hopelessness and had no supportive family had greater odds of suicidal ideation in the past 12 months, (AOR = 1.79; 95% CI = 1.80, 2.97; *p* = 0.024), (AOR = 2.17; 95% CI = 1.30, 3.62; *p* = 0.003), (AOR = 3.89; 95% CI = 1.30, 10.37; *p* = 0.024), and (AOR = 3.19; 95% CI = 1.47, 6.91; *p* = 0.003) respectively (Table 4).

**Table 4:** Logistic regression of factors associated with suicide ideations among students of the University of Zambia and University of Lusaka, Lusaka Zambia July–October 2023.

Variables	Unadjusted regression		Adjusted regression	
	COR (95% CI)	*p*‐value	AOR (95% CI)	*p*‐value
Age (years)				
18–24	Ref	n/a		
25–29	0.81 (0.39, 1.70)	0.578	—	—
Sex				
Female	Ref	n/a		
Male	0.59 (0.38, 0.90)	0.015*	0.61 (0.37, 0.97)	0.036*
Year of study				
First year	Ref	n/a		
Subsequent years	0.60 (0.37, 1.00)	0.032*	—	—
Substance/drug use				
No	Ref	n/a		
Yes	1.83 (1.14, 2.94)	0.012*	1.79 (1.80, 2.97)	0.024*
Depressive symptoms				
Not depressed	Ref	n/a		
Depressed	2.96 (1.83, 4.81)	< 0.001*	2.17 (1.30, 3.62)	0.003*
Hopelessness				
No	Ref	n/a		
Yes	5.06 (1.74, 14.71)	0.003*	3.89 (1.30, 10.37)	0.007*
Supportive family				
Yes	Ref	n/a		
No	4.86 (2.41, 9.79)	< 0.001*	3.19 (1.47, 6.91)	0.003*
Family history of suicide				
No	Ref	n/a		
Yes	1.33 (0.79, 2.25)	0.281	—	—

Abbreviations: AOR, adjusted odds ratio; CI, confidence interval; COR, crude odds ratio; OR, odds ratio; Ref, reference category.

* Statistically significant.

## 4 Discussion

This study investigated the prevalence and factors associated with suicide ideations among students from the University of Zambia and University of Lusaka. The current study reported a prevalence of 33.9% of suicide ideations within the past 12 months. The study revealed that being a male student had reduced odd of presenting suicide ideations than being female. However, students that consumed drugs/alcohol, reported to have had depressive symptoms, were hopelessness about their's future and lacked family social support had significant higher odds of suicide ideations.

This study highlights a concerning prevalence of suicide ideation among university students. This prevalence can be attributed to the increased vulnerability to mental health problems during university years, marked by the responsibility for important decisions and the stress of adjusting to academic and social demands [41, 42, 43]. For numerous students, university attendance can be a stressful and demanding experience. This stress stems from adjusting to a new social environment and facing heightened academic demands. Often, students find it difficult to adapt to the university environment and academic systems. The absence of close social support, often due to living away from home, worsens these challenges for many students [44, 45].

The prevalence reported in this study is consistent with findings from other studies conducted among university students globally. For instance, similar prevalence rates have been reported among student in Pakistan and Ethiopia [46, 47]. Additionally, studies reported comparable rates of suicide ideation at 32.9%, 32.9%, and 31% respectively [44, 45, 48].

However, there is variation in prevalence rates across different studies. Some studies have reported a lower prevalence of suicide ideations among students. These include studies conducted in Ghana, Ireland, Delhi and China which reported a prevalence of 21.3%, 28.5%, 27.2%, and 9.2% respectively [24, 49, 50, 51]. Conversely, studies among college students in Central Indian [52] and among six UK universities [33] reported a higher prevalence rates of suicide ideation at 46.4% and 42.2% respectively.

It's worth noting that the variations in prevalence could be influenced by factors such as cultural differences, sample characteristics, and methodological variations. However, the prevalence reported in the current study is worth addressing and calls for immediate innitatives to improve the mental health of university students in Zambia. Which can be done by in incorporation of mental health programs in higher learning cirriculums by the ministry of higher education in the country [53].

Male students were significantly less likely to have suicide ideations compared to female students. The variance in suicidal behavior between genders may be attributed to disparities in emotional and behavioral issues. Externalizing disorders, more prevalent among males, are linked to suicidal death. Conversely, females tend to experience internalizing disorders such as anxiety and mood disorders, which are associated with suicidal thoughts and behaviors [54]. Additionally, despite males students being less likely to consider suicide, studies have shown that male students are over twice as likely to die by suicide and elevated suicide rates among male students have been consistent since 2000 [55], mirroring trends observed among older adults, where men globally are 2.4 times more likely to commit suicide compared to women [56]. This discrepancy may be attributed to their use of more lethal methods, which reduces opportunities for prevention, intervention, or seeking help for self‐harm at hospitals [57].

The findings reported in this study were similar to finding of a study done in Morocco and Lebanon where male students were less likely to have suicide ideations compared to female students respectively [58]. Additionally, a study done in Uganda found that male students had significantly lesser likely to experience suicide ideations compared to female students [14].

However, a study conducted among first‐year students at Western Cape University found no statistical significant difference in the likelihood of suicide ideation between male and female students [55, 57]. Similarly, another study across 7 Eritrean colleges found that while male students had a higher prevalence of suicide ideation, there was no statistical significant difference in the likelihood of suicide ideation between male and female students [45]. The findings in this study therefore, reecho the need to recognize and address gender differences in suicidal behavior, which can be done by using gender‐specific mental health initiatives.

The use of psychoactive substances increases the risk of suicidal ideation and plans, as well as suicidal attempts in adolescents and young adults [59]. This current study revealed that students that consumed either drugs/alcohol were more likely to have suicide ideations compared to fellow students that did not consume neither drug/alcohol. This relationship may be attributed to the deteriorating effects of psychotropic on one's social relations, ability to develop coping skills and psychosocial skills [41]. Similarly, a study revealed that the consumption of various drugs and alcohol by students across 11 college campuses was statistically associated to suicide ideations [19, 21]. Furthermore, another study revealed that students that were alcohol drinker were more likely to report suicide ideations than non‐drinkers [11, 20]. The findings in this current study were also consistent with a study done among university students in Morocco, which revealed that students who consumed alcohol had a higher odd of suicide ideations compared to the students that did not [58].

However other studies found that alcohol consumption was not a significant predictor of suicide ideations among university students. Suggesting that alcohol related problems exerted effects on the likelihood of both suicide ideation and attempts, the amount students drink is less of a concern for suicidal behavior than are the problems associated with drinking for example failing to meet expectations and experiencing [60, 61].

Furthermore, this study reported a significant association of depressive symptoms and suicide ideations among the students. Students with depressive symptoms had higher odds of suicide ideations which may be because university students are in a developmental period, transitioning from adolescence to adulthood. Which involves adapting to new living conditions as well as new social and academic challenges [57]. This transition makes many students vulnerable to developing mental disorders and when confronted by an adverse life event like a tense campus climate and psychological stress can exacerbate extreme reactions such as suicidal ideations [62]. Research commonly suggests that suicide ideation is often associated with underlying but manageable mental health issues. Studies involving both student and general population samples have found a link between heightened symptoms of anxiety, depression, insomnia, psychosis, and stress and the manifestation of suicidal thoughts [33].

The current study findings were consistent with a study conducted in Brazil [63]. Another study revealed depressive symptom to be associated to suicidal ideation, individuals with depressive symptomatology showed a greater odd chance of engaging in suicidal activity [11, 32]. Another study reported depression as one of the most significant factors associated with of suicide ideations, with students that were depressed being 5.9 times more likely to have suicide ideations [62].

Many studies have found hopelessness to be one variable that has consistently been positively associated with suicidality [64]. In this current study hopelessness was reported to have a significant association with suicide ideations. This was consistent with a study that evaluated hopelessness and suicidal ideation among medical students at Tehran University of Medical Science (TUMS), the study reported hopelessness as independently associated with suicidal ideation among the students [20]. Furthermore, a decade‐long cohort investigation revealed that a specific evaluation of hopelessness conducted at various intervals (baseline, 6, 24, and 48 months) consistently forecasted attempted suicide up to 4–6 years later [27]. The presence of hopelessness has been consistently and autonomously linked to an elevated risk of suicidal ideation, suicide attempts, and suicide‐related mortality, both in prospective and retrospective analyzes [65]. Similarly, studies found that hope among students significantly moderated the relationship between depressive symptoms and suicidal behaviors [63, 65]. Higher levels of hope have been linked to reduced suicidal behavior despite increased depressive symptoms. Similarly, hope has been reported to moderate the association between hopelessness and suicide ideation, elevated hope levels are associated with lower levels of suicide ideation compared to lower hope levels [66, 67].

The current study reported significant higher odds of suicide ideations among students that felt they didn't receive social support from their family. These findings are consistent with other studies that revealed that when a student is given family support, especially parental support, individuals are protected from the onset of suicidal symptoms after experiencing stressful life events [68].

### 4.1 Strengeths and Limitations

This current study used a set of validated tool which gives a great reliability to the results reported. The prevalence reported in this study should be interpreted with caution, as the use of pragmatic convenience sampling may have introduced selection bias and limited the representativeness of the sample. Consequently, the findings may not be generalizable to all university students in Zambia as the primary focus of this study was on individual‐level determinants rather than institutional‐level effects. This sampling approach was necessitated by logistical challenges, as many potential participants were frequently engaged in academic activities, limiting their availability for recruitment. This was an cross‐sectional study and it was unable to examine a causal relationship between suicide ideations and associated risk factors in the study populations. The study recommends that university students should undergo routine screening for suicide ideation and those who screen positive should be recommended for psychological counseling.

## 5 Conclusion

The study examined the prevalence of suicidal thoughts among university students in the past year. Results reported a prevalence of 33.9%, indicating a significant presence of suicidal thoughts among university students at the selected universities. The increased vulnerability during university years, characterized by major decision‐making and responsibilities, contributes to this prevalence. Male students had lower odds of suicidal thoughts compared to females, possibly due to differences in emotional and behavioral issues. Substance use, depressive symptoms, and hopelessness were significant associated with suicidal thoughts. Transitioning to adulthood and facing new challenges makes university students prone to mental health issues. Lack of supportive family relationships was also linked to higher odds of suicidal thoughts. It is important to note that the finding in this study indicate the urgence to address mental health challenges among students, because studies have shown that most suicide attempts are made within a 12 months inceptions of suidice ideations. Therefore continuous assessment of mental well being and frequency of suicide ideations should be prioritized among university students.

## Author Contributions

**Josephine Nkhowani:** conceptualization, methodology, data curation, validation, formal analysis, writing – original draft, writing – review and editing. **Dennis Ngosa:** conceptualization, methodology, formal analysis, writing – original draft, writing – review and editing. **Mwiche Musukuma:** conceptualization, formal analysis, supervision, writing – original draft, writing – review and editing. **Patrick Kaonga:** conceptualization, writing – original draft, writing – review and editing. **Choolwe Jacobs:** conceptualization, supervision, funding acquisition, writing – original draft, writing – review and editing.

## Funding

The authors have nothing to report.

## Conflicts of Interest

The authors declare no conflicts of interest.

## Transparency Statement

The lead/corresponding author Josephine Nkhowani affirms that this article is an honest, accurate, and transparent account of the study being reported; that no important aspects of the study have been omitted; and that any discrepancies from the study as planned (and, if relevant, registered) have been explained.

## Data Availability

The data that support the findings of this study are available on request from the corresponding author. The data are not publicly available due to privacy or ethical restrictions.
